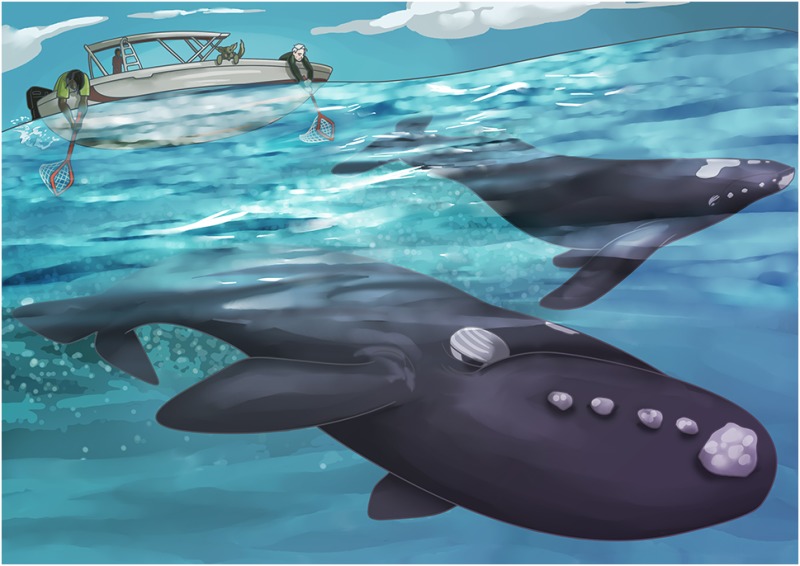# Right whale poo: the key to conserving an endangered species?

**DOI:** 10.1093/conphys/cox063

**Published:** 2017-11-15

**Authors:** Kim Birnie-Gauvin

**Affiliations:** 1 Technical University of Denmark, Vejlsøvej 39, Silkeborg 8600, Denmark

What if a researcher could gather information from animals without having to touch or disrupt them? What about information on an individual’s sex and reproductive state? Apparently, this can now be done simply by collecting a poo sample. Indeed, this is exactly what Corkeron *et al.* have been up to in order to study North Atlantic right whales.

Whales are inherently difficult to sample, given their somewhat cryptic movements, large size, and relatively inaccessible habitats (e.g. diving down to depths of up to 300 m). Researchers working with other aquatic animals, fish for example, can often capture them relatively easily, and physiological parameters can be obtained non-lethally from a blood sample. So, how could one ‘catch’ a whale and flip it over to get some blood? Not really doable.

Luckily, a method was developed and validated a few years ago whereby much of the physiological data obtained from regular blood samples could be obtained from poo samples. Yes, poo. Whale poo is often found floating around where whales are spotted. Dogs can also be used to detect whale feces—yes, some dogs are trained to detect the smell of whale poo! In the best case scenario however, researchers actually witness defecation. In this way, a specific individual can be assigned to its own poo sample. This is especially ideal in the case of North Atlantic right whales. Because, so few individuals remain (<500) that almost all of them are identified and sighted each year.

Hormonal assays can be performed using poo samples, which can provide information about sex hormone levels (such as estrogen, progesterone and testosterone) and stress hormones (such as cortisol). Corkeron *et al.* developed a classification tree that uses data from these hormonal assays to determine the reproductive state of individuals: immature male, mature male, immature female, pregnant female, lactating female or resting female. Because, they have witnessed defecation from 112 individuals—who else in the world could say that?—they can now evaluate how accurate their tree predicts the reproductive state of the whales, given that they had prior knowledge of the reproductive state of each individual. The classification tree was not 100% perfect, but it accurately predicted the reproductive state of individual whales 83% of the time. For comparison, this is equivalent to the accuracy of a range of several medical screening tests! Corkeron and team stress the need to validate their method in every study species however, as each species will likely have different levels and therefore need its own calibration tree.

So, how on earth might poo help us protect these whales? Poo is an honest indicator of physiological state and can be obtained without disturbing an already susceptible population. Therefore, this approach has the potential to identify the proportion of breeding individuals within a population, with huge implications for estimating population size and home ranges. Furthermore, collecting poo samples of unknown individuals may also provide insight on the state of a population exposed to anthropogenic stressors. So, it’s worth it for scientists to get a little stinky to save the whales.

Illustration by Erin Walsh; Email: ewalsh.sci@gmail.com